# 3D-Printing Piezoelectric Composite with Honeycomb Structure for Ultrasonic Devices

**DOI:** 10.3390/mi11080713

**Published:** 2020-07-23

**Authors:** Yushun Zeng, Laiming Jiang, Yizhe Sun, Yang Yang, Yi Quan, Shuang Wei, Gengxi Lu, Runze Li, Jiahui Rong, Yong Chen, Qifa Zhou

**Affiliations:** 1 Department of Biomedical Engineering, University of Southern California, Los Angeles, CA 90089, USA; yushunze@usc.edu (Y.Z.); yizhesun@usc.edu (Y.S.); shuangwe@usc.edu (S.W.); gengxilu@usc.edu (G.L.); runzeli@usc.edu (R.L.); 2 Roski Eye Institute, Keck School of Medicine, University of Southern California, Los Angeles, CA 90033, USA; laiming_jiang@foxmail.com (L.J.); yiquan-xjtu@foxmail.com (Y.Q.); 3 Department of Mechanical Engineering, San Diego State University, San Diego, CA 92182, USA; yyang10@sdsu.edu; 4 Daniel J. Epstein Department of Industrial and Systems Engineering, University of Southern California, Los Angeles, CA 90089, USA; 5 Department of Aerospace & Mechanical Engineering, University of Southern California, Los Angeles, CA 90089, USA; jiahuiro@usc.edu

**Keywords:** 3D-printing, piezoelectric materials, stereolithography, barium titanate, ultrasonic device

## Abstract

Piezoelectric composites are considered excellent core materials for fabricating various ultrasonic devices. For the traditional fabrication process, piezoelectric composite structures are mainly prepared by mold forming, mixing, and dicing-filing techniques. However, these techniques are limited on fabricating shapes with complex structures. With the rapid development of additive manufacturing (AM), many research fields have applied AM technology to produce functional materials with various geometric shapes. In this study, the Mask-Image-Projection-based Stereolithography (MIP-SL) process, one of the AM (3D-printing) methods, was used to build BaTiO_3_-based piezoelectric composite ceramics with honeycomb structure design. A sintered sample with denser body and higher density was achieved (i.e., density obtained 5.96 g/cm^3^), and the 3D-printed ceramic displayed the expected piezoelectric and ferroelectric properties using the complex structure (i.e., piezoelectric constant achieved 60 pC/N). After being integrated into an ultrasonic device, the 3D-printed component also presents promising material performance and output power properties for ultrasound sensing (i.e., output voltage reached 180 mVpp). Our study demonstrated the effectiveness of AM technology in fabricating piezoelectric composites with complex structures that cannot be fabricated by dicing-filling. The approach may bring more possibilities to the fabrication of micro-electromechanical system (MEMS)-based ultrasonic devices via 3D-printing methods in the future.

## 1. Introduction

Researchers from various fields have applied rapidly developed additive manufacturing (3D-printing) technology to their studies, for example, synthesis of biomimetic materials with complex shapes such as nacre and lobster claw, fabrication of micro-electromechanical system (MEMS) devices or piezoelectric medical devices, combining 3D-printing techniques with smart materials for application of 4D-printing, etc. [1,2,3,4,5,6,7]. In fabrication of piezoelectric devices, alignment modes could cause different piezoelectric performance [8]. 3D-printing technology has enabled the produced materials to have isotropic or anisotropic properties for identical layers via the controlled filler alignment [9]. Malakooti et al. illustrated that a 3D-printed nanocomposite could fabricate energy harvesters with high performance when using spatially controlled filler orientation to create embedded nanostructures [10]. Besides, various complex structures could also be fabricated conveniently via 3D-printing methods. Some multiscale structures that were not feasible before can now be constructed, e.g., utilizing an electrically assisted 3D-printing system to fabricate a hierarchical structure with biomimetic nacre-inspired design [11]. In general, the 3D-printing methods are suitable for producing microstructures with comparatively high accuracy and resolution. For cardiovascular stents, a biodegradable stent in mm level with roughly 85–95% accuracy was achieved via a 3D-printing process, which presented an alternative to the conventional laser cutting method [12]. Furthermore, the simplified digital manufacturing process with low cost and high efficiency also illustrated the advantages of the 3D-printing method [13]. At present, 3D-printing methods could be divided into chemical and physical types based on the material forming process. The first type of 3D printing process mainly depends on chemical reactions such as curing agents using optical or thermal resources [14,15]. For example, Kim et al. demonstrated that piezoelectric materials combined with photocurable resin could be cured by visible or invisible lights, and the piezoelectric coefficient was also improved in the printed materials [16,17]. Another type of 3D printing process mainly depends on physical processes such as sintering the material directly using high temperatures provided by devices such as a high-energy laser [18]. Selecting an appropriate 3D-printing method based on the desired functional material and geometric shape is vital for conducting the related research.

As a long-established research field, piezoelectric materials have been widely applied in transducers, ultrasonic motors, sensors, energy harvesters, etc. [19,20,21]. The energy induced by ultrasound or kinetic energy has promising potential in various medical applications, especially in the development of piezoelectric energy harvesters or ultrasonic sensors. For example, a flexible energy harvester with outstanding acoustic, electrical, and mechanical properties was recently implanted wirelessly into eyeballs for the electric stimulation of nerves, in which the piezoelectric part was induced by ultrasound that is harmless to the human body [22]. In general, piezoelectric materials can be classified into the three categories: inorganic materials, organic materials, and composites of both. Some popular examples include lead zirconate titanate (PZT), poly(vinylidene fluoride) (PVDF), and PZT/PVDF composite [23,24]. Currently, PZT is widely utilized in various piezoelectric applications because of its excellent piezoelectric properties (e.g., high piezoelectric coefficient) and ease of manufacturing [23,25]. The fabrication methods of piezoelectric materials include solid-state reaction (SSR), mold forming, casting, dicing-filing techniques, etc. [26,27,28]. For instance, dicing-filling techniques involve implementing parallel cuts on materials via a mechanical dicing saw. Afterwards, the diced material is filled with a polymer in order to create a composite material. Although these traditional fabrication methods can achieve materials with high-density performance, they are tedious with high cost and cannot satisfy the requirements of manufacturing microstructures with complex and meticulous shape design. In MEMS fabrication, stereolithography (SL) has been applied to fabricate MEMS devices with multiple structures [29]. Nonetheless, to overcome the limited two-dimensional device structure, 3D-printing methods have been widely applied to build MEMS devices, such as microfluidic devices or microactuators [30,31]. The advantages of 3D-printing techniques are the ability to fabricate complex micro-shapes, customized design, and relatively low cost. Song et al. reported that resolution of the Mask-Image-Projection-based Stereolithography (MIP-SL) process for layer thickness can reach up to 100 μm [32]. In addition, the development of lead-free piezoelectric materials to replace PZT for environmentally friendly fabrication is a promising trend for future ultrasonic device study [33,34]. Barium titanate (BaTiO_3_, BTO) is the first lead-free perovskite ceramic material that has been developed with excellent dielectric and piezoelectric properties. Previous studies have also demonstrated BTO can be photocured to specific microstructures via 3D-printing methods [16,35,36]. Composite material with a complex structure can also improve material properties (e.g., acoustic and piezoelectric properties) [19]. Moreover, the honeycomb structure could be fabricated as a composite material by introducing low-permittivity epoxy. As a result, the voltage coefficient (*g*_33_ = *d*_33_/*ε*) can be augmented by lowering the permittivity, effectively improving the sensitivity of the piezocomposite to sense ultrasound [22]. Kim et al. also showed the 3D-printed honeycomb structure could be optimized to achieve better material properties, such as excellent electric properties, mechanical strength, and larger specific surface area [37,38,39]. Inspired by this work, we further exploited the use of 3D-printing techniques to fabricate piezoelectric materials with complex structure design to understand the full potential of 3D-printing for ultrasound applications.

Herein, using a selected 3D-printing method, we present the study of using the Mask-Image-Projection-based Stereolithography (MIP-SL) process to fabricate lead-free piezoelectric material with a complex honeycomb structure. Relevant piezoelectric properties of the 3D-printed samples were studied to understand the effectiveness of the 3D-printing approach. To further test the material performance of ultrasound sensing, an ultrasonic device was integrated. Comparing with other fabrication methods, the MIP-SL approach simplifies the fabrication process and enables piezoelectric materials with complex structures to be designed for ultrasonic devices. The test results show the fabricated piezoelectric structures possess adequate ferroelectric and piezoelectric properties. The fabricated honeycomb composite structure optimized the piezoelectric properties and reduced the acoustic impedance. Such benefits could be further investigated as a potential wireless power source. Thus, the MIP-SL method offers researchers a good fabrication tool that enables the design of various structure shapes so related piezoelectric devices can have satisfactory piezoelectric performance.

## 2. Materials and Methods

**Figure 1:**
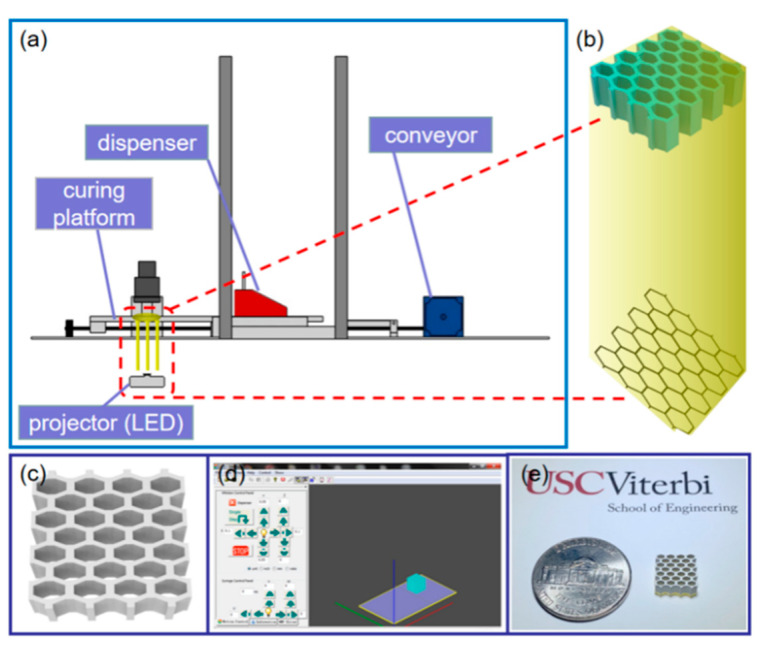
(**a**) Mask-Image-Projection-based Stereolithography (MIP-SL) system to print green parts. (**b**) Sliced 2D pattern of a 3D model projected by a digital light projector. (**c**) Computer-aided design model of the printed sample with honeycomb structure. (**d**) Graphical user interface of the MIP-SL system developed in-house. (**e**) Picture of the green part fabricated using the MIP-SL system.

Barium titanate particles (BaTiO_3_, Sigma-Aldrich Co., Saint Louis, MO, USA) of 50 wt%, 60 wt%, and 70 wt% were mixed with 50 wt%, 40 wt%, and 30 wt% photocurable resin (SI500, EnvisionTec Inc., Ferndale, MI, USA) respectively, to create the composite slurry via ball milling in pulverisette (model LC-105-4, Gilson company. Inc., Lewis Center, OH, USA). The highest concentration of piezoelectric powder for successful printing can reach up to 70 wt%. The BaTiO_3_ and resin mixtures went through ball milling at 200 rpm for 30 min to become a homogeneous slurry. The slurry was then vacuumed for 10 min to remove all the inside air bubbles. Afterwards, the mixture of prepared slurry was spread on a building platform of the MIP-SL system (Figure 1a) using a blade. The slurry was evenly tape-casted on the platform by the blade. The dispersed slurry layer was then transmitted to the visible light area (wavelength of 405 nm) defined by a LED-based digital light projector using a linear stage to induce the photocuring process (Figure 1b). With several experiments, the doctor blade height was set to be 100 µm, while the exposure time was optimized as 37 s per layer, and the thickness of each layer could be fabricated equally as 30 μm. The model built from Solidworks (Figure 1c) was sliced into two-dimensional (2D) images using an in-house developed control system (Figure 1d). After repeating the layer-based fabrication process, the green part of the model was fabricated (Figure 1e). Based on it, the printed samples were first debinded to remove the photocured resin in the green parts of the structures. The debinded samples were then sintered at 1350 °C for 4 h to create dense ceramic parts, which converted the debinded sample into a fully sintered ceramic part with a dense structure between each layer [36,40,41].

**Figure 2:**
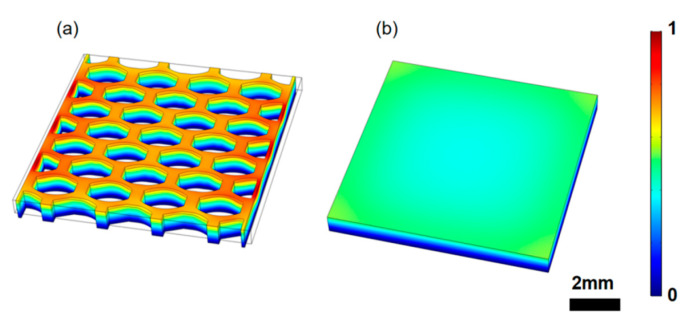
The simulated piezoelectric potential distribution inside the samples with (**a**) honeycomb structure and (**b**) solid brick structure.

Both the fabricated green parts (before debinding) and the sintered samples were observed and compared under a microscope (SZ61, Olympus, Tokyo, Japan). The density of the sintered samples was measured using the Archimedes method, and the sintered samples were poled under the condition of 2 kV/cm at 25 °C for 30 min. The capacitance and impedance spectrums were characterized by an impedance analyzer (,Agilent 4294A, Santa Clara, CA, USA). The polarization-electric field (P-E) hysteresis loop of the samples was measured by a ferroelectric measuring system (Hysteresis Version 3.1.1, Radiant Technologies, Inc., Albuquerque, NM, USA). The piezoelectric coefficient *d*_33_ was characterized by a *d*_33_ m (YE2730A, APC international, Ltd., Mackeyville, PA, USA). Moreover, simulations for the ultrasound-induced piezoelectric potential were conducted with finite element analysis software (Comsol Multiphysics 5.3a, COMSOL Inc., Stockholm, Sweden), as illustrated in Figure 2.

An accordingly designed ultrasonic device was constructed using the 3D-printed piezoelectric structure after the debinding and sintering procedures in order to further investigate the performance of the 3D-printed sample. Each hole of the sintered sample with honeycomb structure design was filled with epoxy (Epo-Tek 301, Billerica, MA, USA) in order to gain a composite material sample. After curing the filled epoxy, the top and bottom sides of the sintered sample were sputtered with Au/Cr electrodes (NSC-3000 Sputter Coater, Nano-Master Inc., Austin, TX, USA) and connected with copper wires. Liquid Rubber (Ecoflex 00-30, PA, USA) was mixed to fill the sample to construct the device. The output voltage amplitudes were measured for different input voltages using the built ultrasonic system with the integrated ultrasonic transducer to achieve the ultrasound sensing.

## 3. Results and Discussion

### 3.1. Simulation Performance

In this work, the sample was placed in deionized water, and the holes of the honeycomb structure were filled with insulating epoxy. The mechanical deformation induced by ultrasound waves occurred throughout the whole structure. During the polarization process, charge dipoles were produced in the ceramic structure of the piezoelectric composite. When the structure of the sample was mechanically deformed, piezoelectric potential emerged between the top and bottom electrodes. Induced electrons flowed through the external structure so that the generated piezoelectric field could be balanced. Electrons accumulated at the bottom electrode, which produced a signal of voltage and current. Thus, the ultrasonic energy could be converted by the piezoelectric sample into an output of piezoelectric potential.

To illustrate the advantages of printed ceramics, a finite element analysis was employed to demonstrate the ultrasound sensing process. Specifically, the performance of the composite honeycomb structure (Figure 2a) was compared with a solid single crystal structure (Figure 2b). The parameters of the sample were selected from the COMSOL material library. The density and piezoelectric constant were corrected according to the test results in order to mimic real conditions of the sintered sample. Both samples were 8.5 mm × 8.5 mm × 0.8 mm, and the simulation had a density of 5.96 g/cm^3^, a piezoelectric constant of 60 pC/N, a Young’s modulus of 67 GPa, and a 0.33 Poisson’s ratio. The epoxy in the composite structure had a density of 1.67 g/cm^3^, a Young’s modulus of 3.96 GPa, and a 0.33 Poisson’s ratio. The bottom of samples was mechanically fixed and connected to the ground. An ultrasound field at 1.6 MHz with the same intensity transmitting from top to bottom was applied to both samples. The distribution of ultrasound-induced piezoelectric potentials was calculated and plotted in Figure 2. The color bar represents the normalized ultrasound-induced electric potential distribution. It is shown that the generated piezoelectric potential in the honeycomb structure was about twice that of the solid brick structure. Thus, the honeycomb structure can perform better than solid structures. The application of the proposed honeycomb structure for high-sensitivity ultrasound sensing deserves to be studied further.

### 3.2. Characterization of Green Parts and Sintered Samples

**Figure 3:**
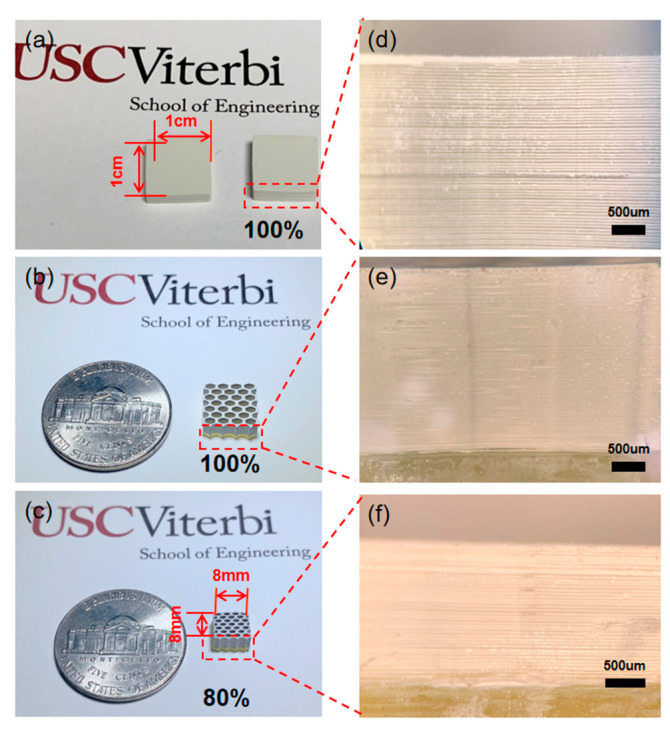
(**a**) Green part sample with brick structure. (**b**) Green part sample with honeycomb structure in 100% size ratio. (**c**) Green part sample with honeycomb structure in 80% size ratio. (**d**–**f**) Layer details of the green parts under a microscope.

BaTiO_3_ green part samples with a brick structure, using 100% and 80% ratio sizes of a honeycomb structure, were built via the MIP-SL system (Figure 1a). Figure 3a–c shows images of the 3D-printed piezoelectric green parts. The size ratio refers to the proportion of a different structure’s basal side length to the basal side length with a fixed basal area of 1 cm × 1cm. One hundred percent size ratio samples gained the designed shape with the basal area size of 1 cm × 1 cm, while 80% size ratio samples obtained shapes with the basal area size of 0.8 cm × 0.8 cm. Each hole of the structure (100% ratio) was defined by a hexagon with side length 800 μm, and the wall thickness of the structure was 450 μm. Figure 3d–f shows the layer details of the samples under the microscope with a scale bar of 500 μm. Each sample had a dense structure made by the MIP-SL system. However, due to the limited Z curing depth and XY resolution of the MIP-SL system, layers of the 80% size ratio sample had small gaps and were not perfectly dense. Furthermore, the brick structure sample showed obvious cracking during the sintering process due to the internal stress of the brick structure. Hence, the small holes of honeycomb structure were then filled with epoxy after the sintering process to construct the required piezoelectric composites for further study.

**Figure 4:**
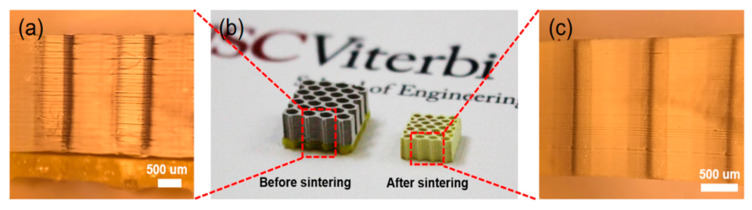
(**a**) Layer details of the sample before sintering. (**b**) Size comparison of the samples before and after sintering. (**c**) Layer details of the sample after sintering.

Figure 4b shows that the whole size of the sample (100% ratio) became smaller after sintering. The sintered sample size became 8.5 mm × 8.5 mm. In particular, comparing Figure 4a,c, few gaps existed after the photocured resin was removed from the structure during the debinding process. The layered structures became denser after sintering. To compare with pure BaTiO_3_, Table 1 shows the density of BaTiO_3_ ceramic samples measured using Archimedes’ principle before and after sintering [42]. The density of the printed samples after sintering increased significantly, indicating the 3D-printed BaTiO_3_ ceramics converted to dense bodies during the sintering process.

### 3.3. Device Fabrication

**Figure 5:**
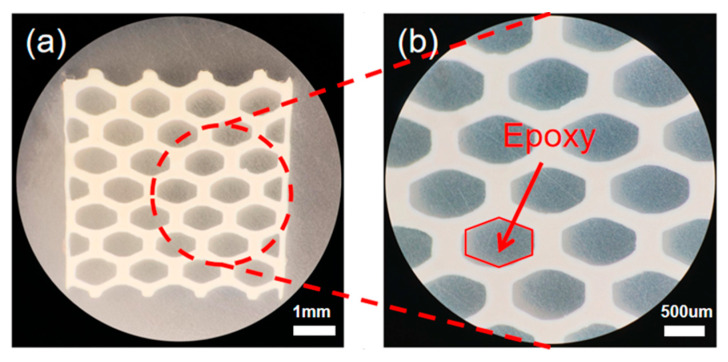
(**a**) Sintered sample filled with epoxy resin under a microscope with a scale bar of 1 mm. (**b**) Detail of the sample under the microscope with a scale bar of 500 μm.

**Figure 6:**
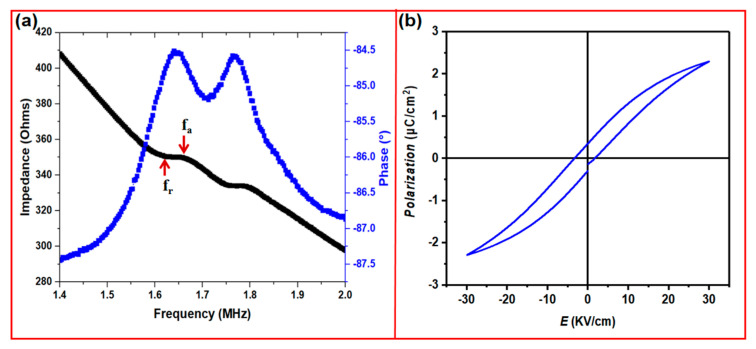
(**a**) Impedance and phase angle spectrum of the sputtered sample. (**b**) Polarization-electric field (P-E) hysteresis loop of the sputtered sample.

An ultrasonic device was constructed using the sintered BaTiO_3_ ceramic sample fabricated with the MIP-SL technique. Figure 5a,b illustrates the sintered sample filled with epoxy under the observation of a microscope. The epoxy was filled into the holes of the sample. After curing the filled epoxy at 40 °C for 4 h, the sample was ground to 800 μm in thickness. Then, the top and bottom sides of the sample were sputtered with Au/Cr electrodes for electrical connection. The sputtered sample was poled under a DC electrical field of 20 kV/cm at 25 °C for 30 min. Figure 6a shows the spectrum of impedance and phase angle of the poled sample, which was measured using an impedance analyzer (Agilent 4294A, Santa Clara, CA, USA). The electromechanical coupling coefficient (*k_t_*) of the piezoelectric material can be defined as Equations (1) and (2) [36].

(1) $$k_{t}=\sqrt{\frac{Mechanical\;energy\;stored}{Electrical\;energy\;applied}}$$

(2) $$k_{t}\sqrt{\frac{\pi fr}{2fa}\times cot\frac{\pi fr}{2fa}}$$

where *f_r_* is resonant frequency, and *f_a_* is anti-resonant frequency. Figure 6a shows that *f_r_* and *f_a_* are 1.60 MHz and 1.66 MHz, respectively. Thus, *k_t_* is calculated to be about 31.1%. The coupling coefficient of the 3D-printed sample was measured three times using the same method, and the measured coefficient value did not have a significant difference (it ranged from 31.1%–31.5%). The piezoelectric constant is 60 pC/N. The results show that the 3D-printed BaTiO_3_ ceramics gained the piezoelectric property.

To characterize the ferroelectric properties, the polarization-electric field (P-E) hysteresis loop was measured. Figure 6b shows the P-E curves of the sputtered samples, which were measured under an electric field with an intensity of 30 kV/cm. The P-E curve clearly demonstrates a typical ferroelectric hysteresis loop. Furthermore, it displays the remnant polarization (*P_r_*) was 0.346 μC/cm^2^ and the maximum polarization (*P_max_*) was 2.29 μC/cm^2^. The coercive field (*E_c_*) was 3.645V/cm. In summary, our experimental results illustrate that the 3D-printing method can produce piezoelectric ceramics with complex structures to achieve desired ferroelectric properties.

**Figure 7:**
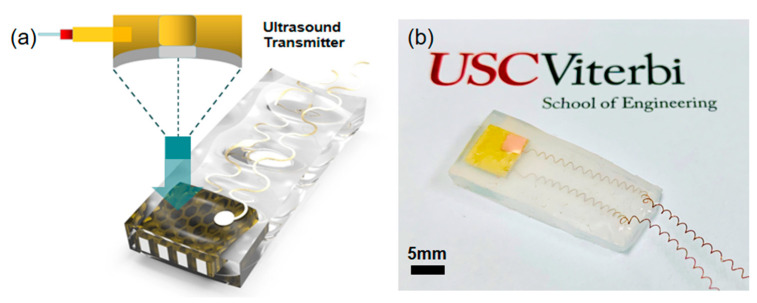
(**a**) Schematic and design of the ultrasonic device. (**b**) Optical image of the fabricated device.

After testing the properties of the 3D-printed sample, in order to test the material performance, the sputtered sample was further utilized to fabricate an ultrasonic device for ultrasound wave sensing. Figure 7a shows the schematic design of the device. Both sides of the sputtered device were connected with copper wires, and then the combined components were encapsulated with Ecoflex resin to form the final ultrasonic device (Figure 7b). Finally, an ultrasound test system consisting of a 1 MHz ultrasound transmitter, a function generator, an amplifier, and an oscilloscope was used to evaluate the ultrasound sensing performance of the device [22].

**Figure 8:**
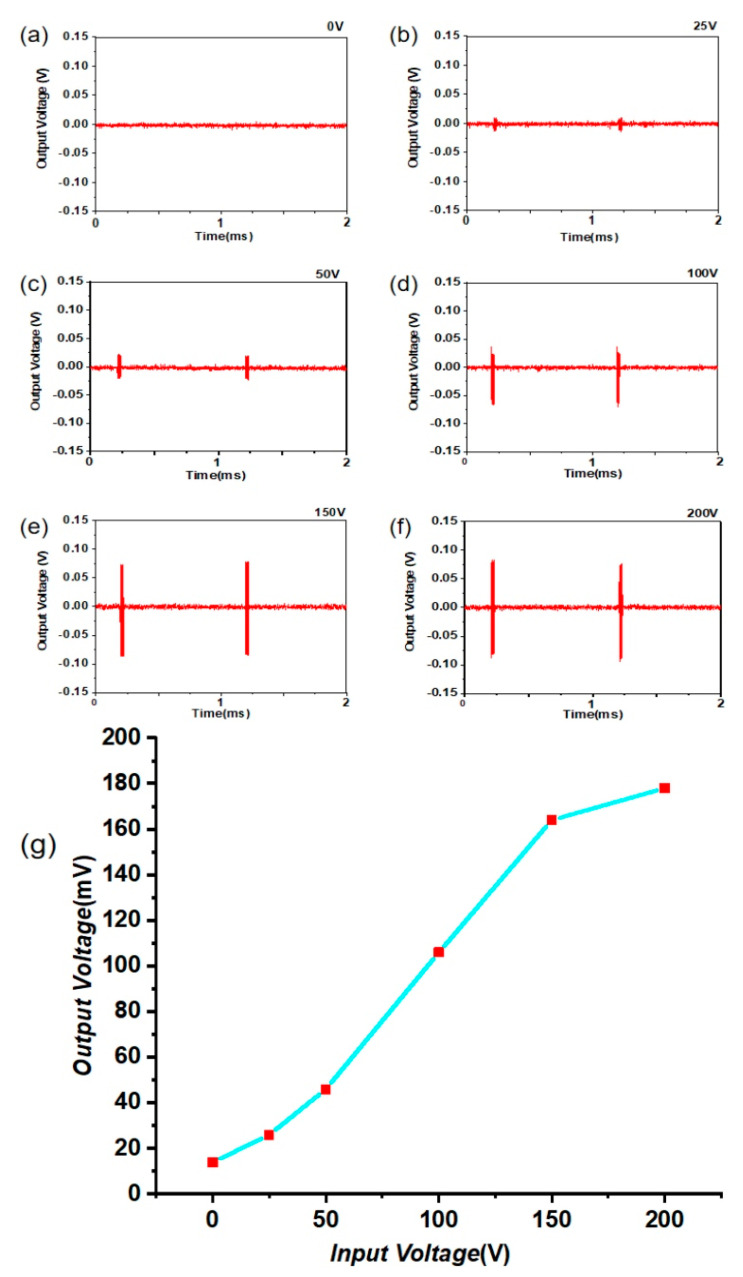
Output voltage amplitudes of the device over time for different input voltages of (**a**) 0 V; (**b**) 25 V; (**c**) 50 V; (**d**) 100 V; (**e**) 150 V; and (**f**) 200 V; (**g**) Trend of the output voltage.

To characterize the sensing capability of the manufactured ultrasonic device, the ultrasound transmitter generates ultrasound under various input voltages at a frequency of 1 MHz, and then the ultrasound directly propagates to the area of the device. The 3D-printed device receives the transmitted ultrasound waves and converts them into electricity through the piezoelectric effect. The input voltage amplitudes of the device for various input voltages from 0 V to 200 V are shown in Figure 8a–f, which show the output power of the device with the 3D-printed sample. The oscilloscope was set at an internal impedance of 1 MΩ. Interestingly, as shown in Figure 8g, by increasing the input voltage of the system, the amplitude of the output voltage demonstrated a sharp increase at first, then, after the input voltage was over 150 V, the amplitude increased less. Finally, the maximum output voltage reached 180 mVpp without any further amplification. Correspondingly, the voltage efficiency was about 0.1% and the output power about 9 nW. The test results demonstrate that the output voltage could be induced during ultrasonic propagation from the device using the piezoelectric structure fabricated via the 3D-printing method. Additionally, Table 2 summarizes essential performance parameters of the ultrasonic device with honeycomb structure. Although the output power was not high enough, it can be applied by devices with low power. In future investigations, the output power will be further optimized by improving mechanical, electric, and acoustic properties of the printed material [43,44,45].

## 4. Conclusions

In summary, an ultrasonic device with honeycomb structure design was fabricated using a 3D-printing method (Mask-Projection-based stereolithography) from a composite slurry of mixed BaTiO_3_ powder and photocurable resin. One designed complex structure (honeycomb structure) was faithfully fabricated using the 3D-printing method. Fabricated layers observed using a microscope demonstrated dense structures after the sintering process. The 3D-printed sample achieved prospective piezoelectric and ferroelectric performance. The capability of the 3D-printing process to fabricate piezoelectric ceramics with complex structures that may lead to the increase of piezoelectric efficiency has also been demonstrated. Furthermore, the sintered 3D-printed sample was integrated into an ultrasonic device for ultrasound sensing. The result of the output voltage amplitudes showed comparable ultrasound sensing performance of the 3D-printed sample. The advantages of utilizing 3D-printing to fabricate MEMS devices with complex structures for biomedical applications need to be further explored in the future.

## Figures and Tables

**Table 1:** The density of BiTO3 in different conditions.

Characteristics	Before Sintering	After Sintering	Pure BaTiO_3_ [42]
Density (g/cm^3^)	1.21	5.96	6.02

**Table 2:** Performance parameters of the ultrasonic device.

Characteristics	Ultrasonic Device with Honeycomb Structure
*d*_33_ (pC/N)	60
Ec (kV/cm)	3.645
P_max_ (μC/cm^2^)	2.29
Thickness (μm)	800
Density (g/cm^3^)	5.96
Resonant frequency (MHz)	1.6
Output voltage (mVpp)	180
Output power (nW)	9
